# The influence of neoadjuvant chemotherapy on gastric cancer patients’ postoperative infectious complications: What is the negative role played by the intestinal barrier dysfunction?

**DOI:** 10.18632/oncotarget.14758

**Published:** 2017-01-18

**Authors:** Zhiliang Wei, Bin Tan, Shougen Cao, Shanglong Liu, Xiaojie Tan, Zengwu Yao, Na Yin, Jiante Li, Dongfeng Zhang, Yanbing Zhou

**Affiliations:** ^1^ Department of General Surgery, Affiliated Hospital of Qingdao University, Qingdao, China; ^2^ Department of Hepatobiliary Surgery, Affiliated Hospital of Qingdao University, Qingdao, China; ^3^ Department of General Surgery, Yantai Yuhuangding Hospital, Yantai, China; ^4^ Department of Histology and Embryology, Binzhou Medical University, Yantai, China; ^5^ Department of Epidemiology and Health Statistics, Qingdao University Medical College, Qingdao, China

**Keywords:** neoadjuvant chemotherapy, gastric cancer, postoperative infectious complications, intestinal barrier dysfunction, intestinal microbiology

## Abstract

Evidence has shown that neoadjuvant chemotherapy (NACT) is correlated with patients’ overall postoperative complications. But investigations on relationship between NACT and postoperative infectious complications, which is closely linked to intestinal barrier damage, were scanty. Accordingly, 90 patients with advanced gastric cancer were included in this study. The differences in postoperative infectious complications were determined between NACT group in which patients received NACT before surgery and SURG group in which received surgical treatment immediately after diagnosis. The damage of mechanical structure of intestinal barrier was assessed by hematoxylin and eosin staining, transmission electron microscopy, and immunohistochemistry. Mucosal microbiota changes were determined by using a 16S rRNA gene sequencing approach. Results showed that the incidence of postoperative infectious complications were significantly higher in the NACT group. Tight junctions were disrupted, and claudin-1, ZO-1 and occludin were down-regulated in patients with infectious complications in overall compared with those without. And similarly, the patients in the NACT group also showed damaged intestinal barrier compared with those in SURG group. Besides, the total diversity of mucosal related bacteria was decreased and relative abundance of some probiotics, such as *Bifidobacterium*, *Faecalibacterium* and *Ruminococcus*, was reduced in the NACT group as well. In conclusion, our study identifies a higher incidence of postoperative infection in gastric cancer patients who underwent NACT treatment, and these changes might be caused by a significant damage in the intestinal barrier as well as reduced probiotics.

## INTRODUCTION

Neoadjuvant chemotherapy (NACT) have been employed to improve the survival rate for cancer patients. [1–3] And nowadays, most RCT (randomized control trial) focus on the long-term effects that patients can benefit from NACT rather than the side effects it brings in the short term [3, 4]. Although many studies, which engaged in proving the safety of using NACT, exhibited no significant difference in overall postoperative complications [5–7], few papers mentioned that postoperative infections were more common in patients who underwent NACT [8]. Given that chemotherapy has been known to cause bone marrow suppression [9] and intestinal barrier structural damage [10] in animal models which might raise the risk for infection, so we hypothesize that intestinal barrier injuries are the key point between preoperative chemotherapy and postoperative infections.

In this paper, we designed a cohort study which enrolled 90 patients to exam the specific correlation between NACT and patients’ postoperative infection-related complications. The status of the intestinal barrier was evaluated between patients with and without infectious complications in overall and each group (SURG and NACT). Histological examination, electron microscope analysis and expression of claudin-1, ZO-1 (zonula occludens-1 protein) and occludin proteins were used to determine the mechanism of structural damage. Moreover, mucosal related microbiota, which functions as another important barrier structure was explored by 16S rDNA sequencing analysis. Hopefully, this approach may reveal the pathological process of how preoperative chemotherapy could affect patients’ postoperative infections.

## RESULTS

### Infection-related complications between the NACT and SURG group

103 patients were recruited in our study. Among them, 13 patients, whose cancer proved to be pathologic stage T4b/N+M0 during surgery and received combined multiple organ resection, were excluded according to the exclusion criteria. Therefore, eventually 60 patients were assigned to the SURG group, and 30 patients were assigned to the NACT group. The clinical characteristics of the patients in the two groups were compared in Table 1. There were no significant differences with regard to age (*P* = 0.818), gender (*P* = 0.393), BMI (*P* = 0.091), NRS2002 (*P* = 0.333), ASA score (*P* = 0.528), Resection range (*P* = 0.113), intraoperative blood transfusion (*P* = 0.488), fever time (*P* = 0.072) and Lauren's classification (*P* = 0.869) between the NACT and SURG groups. While operative time (*P* = 0.009), antibiotics usage (*P* = 0.004), hospital stay after operation (*P* = 0.012), tumor pathologic stage (*P* = 0.036) and total costs (*P* = 0.025) were higher in NACT group. Therefore, it suggested that the baseline of the two groups was quite similar, but the incidence of postoperative complications might be different.

**Table 1:** The clinicopathological characteristics of enrolled patients

	NACT	SURG	*P* value
Age (year)	59.4±9.1	60.0±10.8	0.818
Gender			0.393
Male	24	43	
Female	6	17	
BMI (kg/m^2^)	24.8±3.2	23.6±2.9	0.091
NRS2002	2.3±0.7	2.5±1.0	0.333
ASA			0.528
I	0	3	
II	22	43	
III	8	13	
IV	0	1	
Resection range			
Total	10	11	0.113
Sub-total	20	49	
Operative time (min)	195.7±44.5	172.4±35.8	0.009*
Intraoperative blood transfusion (ml)	163.3±122.4	144.3±121.6	0.488
ICU stay (day)	0.6±1.8	0.1±0.4	0.093
Antibiotics usage (day)	4.3±5.9	0.8±2.7	0.004*
Fever time (day)	5.9±4.7	4.5±2.5	0.072
Hospital stay after operation (day)	12.3±9.2	8.8±3.6	0.012*
Total costs (dollars)	14356.7±6829.0	11294.3±2994.0	0.025*
Tumor pathologic stage			0.036*
II	5	23	
III	25	37	
Lauren's classification			0.869
Intestinal type	9	17	
Diffuse type	21	43	

*: Statistically significant P<0.05; BMI: Body mass index; ASA: American society of anesthesiologists scores; NRS2002: nutrition risk screening 2002.

The differences of postoperative infectious complications, graded by Clavien-Dindo classification (Table 2), were compared between the two groups. The result exhibited that the morbidity rate of infectious complications was elevated in the NACT group compared with the SURG group (43.3% *vs*. 13.3) and the incidence was significantly higher in patients who underwent NACT (Table 3).

**Table 2:** Infectious complications based on Clavien-Dindo classification

Clavien-Dindo classification	Complications				
	Bacteremia	Urinary tract infection	Pneumonia	Incisional SSI	Organ or space SSI
I	1 (0/1)		2 (1/1)	3 (3/0)	
II	2 (2/0)	1 (1/0)	5 (3/2)	1 (1/0)	4 (1/3)
III					1 (1/0)
IV			1 (0/1)		

In the brackets: Cases in NACT group/Cases in SURG group.

**Table 3:** The difference of infectious complications between NACT and SURG groups

	NACT	SURG	*P* value^#^
Clavien-Dindo classification			0.002*
0	17	52	
I	4	2	
II	8	5	
III	1	0	
IV	0	1	

#: Mann-Whiteny test,

*: Statistically significant *P* < 0.05

### Histopathological examination

**Figure 1:**
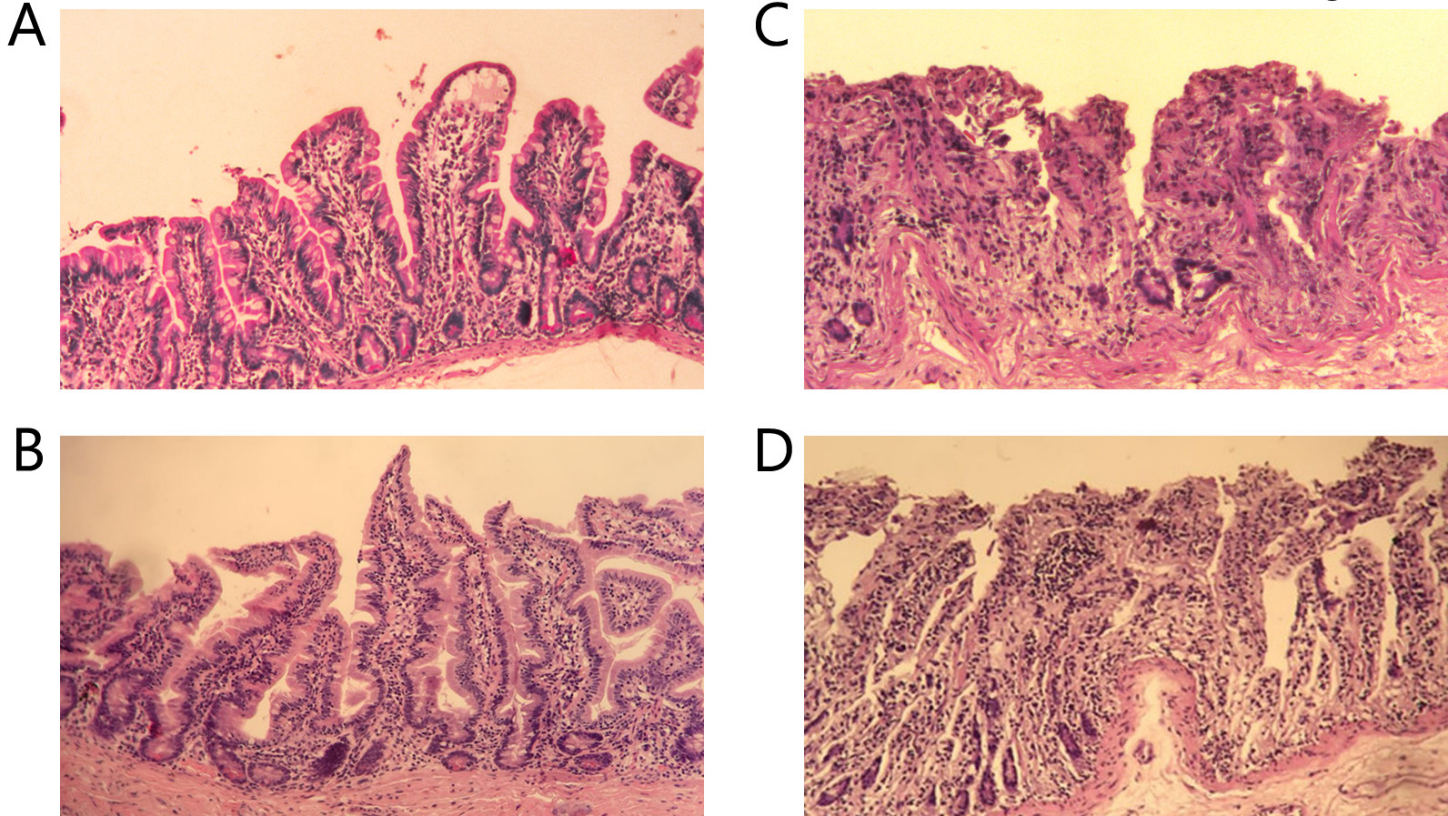
Histopathology of ileum sections The intestines of patients without infectious complications **A**. and those in SURG group **B**. showed normal villous architecture and glands, with no obvious destruction, while the intestinal mucosa injuries of patients with infectious complications **C**. and those in NACT group **D**. were shown with massive epithelial lifting down the sides of the villi and ulceration at the villous tips. (×200).

The histopathological findings showed that the villi of the patients without infectious complications was intact and there was no epithelial disruption. While in the patients with infectious complications, mucosal atrophy could be found and destruction of the villi were evident when contrasted with those without, the same pattern was also observed between the SURG group, which exhibited lower incidence of infectious complications, and NACT group (Figure 1). Chiu's grade scores are shown in Table 4.

**Table 4:** Grade of intestinal mucosal injury in different groups

	Infection	Non-infection	*P* value	NACT group	SURG group	*P* value
Chiu's score			<0.001*			0.002*
≤ 1	9	64		19	54	
≥ 2	12	5		11	6	

*: statistically significant *P* < 0.05

Abbreviation: Infection: patients with infectious complications in overall, Non-infection: patients without infectious complications in overall

### Differences in ultrastructural characteristics

**Figure 2:**
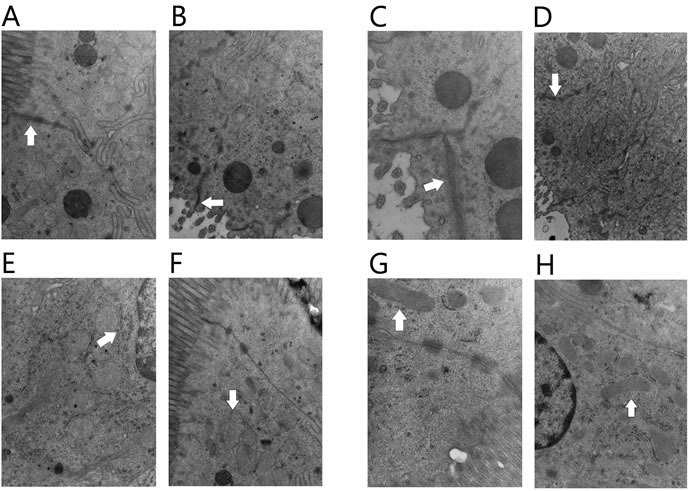
Transmission electron microscopy of the intestine mucosal The tight junction was intact in patients without infectious complications **A**. and those in SURG group **B**. (×15000); while the tight junction was unclear and obviously disrupted in patients with infectious complications **C**. and those in NACT group **D**. (×15000). Organelles were almost normal in patients without infectious complications **E**. and those in SURG group **F**. (×20000), while organelles were swollen in patients with infectious complications **G**. and those in NACT group **H**. (×20000).

The ultrastructure of intestinal mucosa in patients with infectious complications and those in NACT group were significantly changed when compared to the patients without infectious complications and those in SURG group. The structure of tight junction was not clear and disrupted, while desmosome connection was dissolved. The mitochondrial matrices were swollen and deformed along with broken cristae (Figure 2).

### Expression of the TJ protein

**Figure 3:**
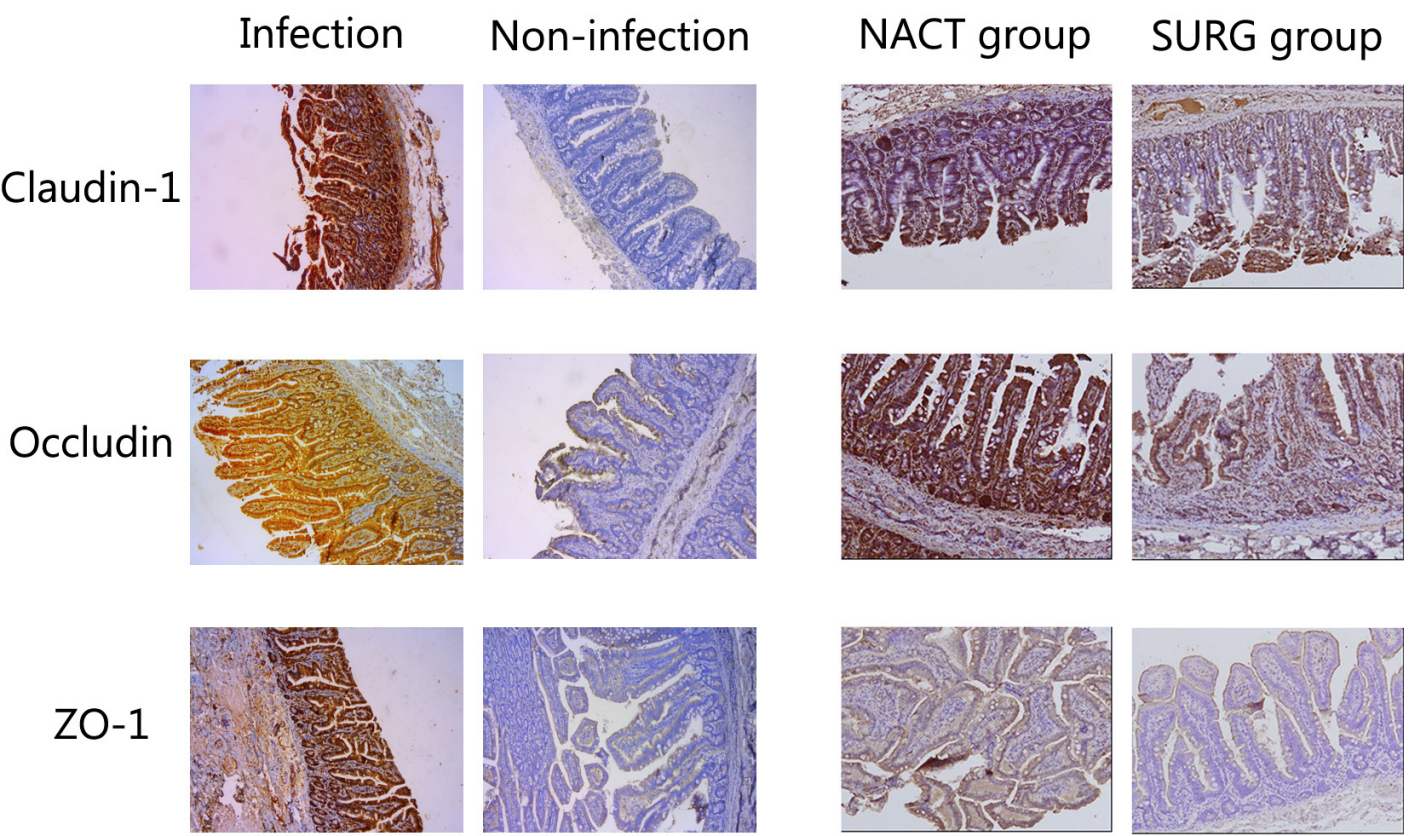
Immunohistochemistry was used to analysis the expression of Claudin-1, Occludin and ZO-1 in tissues, and they all showed higher expression in patients without infectious complications and those in SURG group than patients with infectious complications and those in NACT group respectively (×100)

Immunohistochemistry was used to detect the differences of claudin-1, occludin and ZO-1 expression (Figure 3). By comparing immunohistochemical scores of tissues, we observed that claudin-1, occludin and ZO-1 expression were lower in patients with infectious complications than those without. Likewise, patients in the NACT group also showed lower expression of TJ proteins than those in the SURG group. (Table 5)

**Table 5:** Correlations of different groups with IRS of Claudin-1, Occludin and ZO-1 expression

Group	Claudin-1 Mean SD *P* value			Occludin Mean SD *P* value			ZO-1 Mean SD *P* value		

Infection	3.571	1.630	<0.001*	1.952	1.532	0.001*	3.524	1.965	<0.001*
Non-infection	5.710	2.340		3.478	1.915		6.145	2.580	
NACT	4.200	1.937	0.004*	1.967	1.068	<0.001*	4.333	2.322	0.002*
SURG	5.717	2.415		3.700	2.022		6.183	2.610	

*: statistically significant *P* <0.05

Abbreviation: Infection: patients with infectious complications in overall, Non-infection: patients without infectious complications in overall

### Diversity and structural differences of the mucosa-associated microbiota between the NACT and SURG group

Libraries of 16S rRNA V4 region amplicon sequences from mucous samples were sequenced. After filtering out the OTUs with very low counts, 13,770 OTUs in all samples were identified with dissimilarity level of 3%.

**Figure 4:**
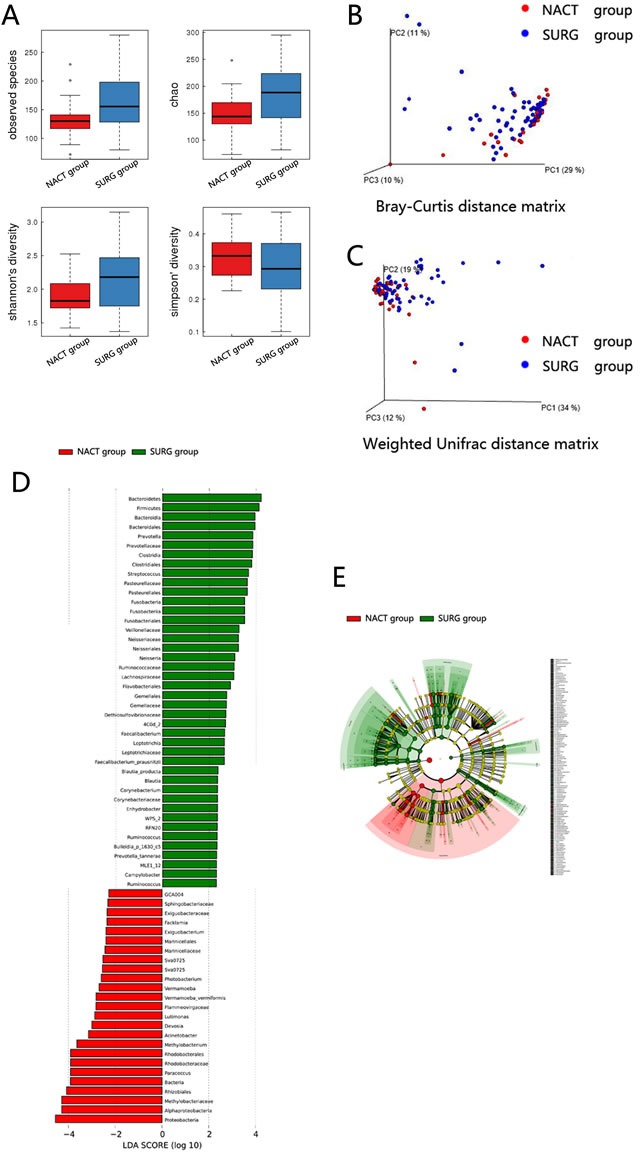
Diversity and structural changes of the tumor microbiota between the NACT group (*n* = 90) and SURG group (*n* = 30) **A**. Alpha-diversity distances calculated using phylotype relative abundance measurements among groups. Principal coordinates analysis (PCoA) scores plot of Bray-Curtis distance matrix **B**. and weighted Unifrac distance matrix **C**. based on the relative abundance of OTUs (97% similarity level). Each symbol represents a sample. Colors represent different groups. **D**. Histogram of the LDA scores for differentially abundant genera between NACT group and SURG group. Cladogram was calculated by LefSe, and displayed according to effect size. **E**. Taxonomic representation of statistically and biologically consistent differences. Differences are represented by the color of the most abundant class (red indicating control group, green tumor group and yellow non-significant). The diameter of each circle's diameter is proportional to the taxon's abundance.

The Good's coverage value was over 99% for each group. Estimators of community richness, diversity and evenness were calculated based on the OTUs among groups (Figure 4A). Marked differences in observed species index (132 ± 33 *vs*. 164 ±47, *P* = 0.001), Chao diversity index (147 ± 36 *vs*. 184 ±53, *P =* 0.001) and Shannon diversity index (1.88 ± 0.25 *vs*. 2.15 ±0.46, *P* = 0.008) were detected between the NACT group and SURG group which demonstrated that both microbial diversity and evenness were significantly decreased in the NACT group.

For beta diversity analysis, Bray-Curtis distance matrix and weighted Unifrac distance matrix were adapted to analyze and compare the microflora and compositions between these two groups based on the OTUs (97%similarity). Subsequent results of principal coordinates analysis showed that the bacterial community composition was significantly different between groups. The first three principal component scores were 29%, 11%, 10% and 34%, 19%, 12% of explained variance respectively (Figure 4B and 4C).

The dominant phyla of both groups were *Proteobacteria* (86.7%-95.2%), *Firmicutes* (2.2%-5.5%), *Bacteroidetes* (2.2%-5.9%) and *Actinobacteria* (0.4%-0.6%). When comparing the relative abundance of phyla between the groups, we found that higher abundance of *Proteobacteria* was exhibited in the NACT group than in the SURG group (94.6% *vs*. 87.5%, FDR = 0.004), while *Firmicutes* (2.3% *vs*. 4.9%, FDR = 0.016), *Bacteroidetes* (2.2% *vs*. 5.5%, FDR = 0.0046) and *Actinobacteria* (0.37% *vs*. 0.54%, FDR = 0.043) were lower in the NACT group.

The microbial composition was different at the genus level. Significantly higher abundance of *Prevotella* (0.6% *vs*. 2.1%, FDR = 0.004), *Methylopila* (0.3% *vs*. 1.7%, FDR = 0.013), *Bifidobacterium* (0.09% *vs*. 0.27%, FDR = 0.047), *Faecalibacterium* (0.04% *vs*. 0.13%, FDR = 0.03) and *Ruminococcus* (0.02% *vs*. 0.04%, FDR = 0.007) were shown in the SURG group. While *Methylobacterium* (1.06% *vs*. 0.16%, FDR = 0.004) was relatively more abundant in the NACT group.

The main differences in taxa between the two groups were shown by linear discriminant analysis (LDA) and effect size measurements (LEfSe) in Figure 4D. The cladogram representation and the predominant bacteria of the microbiota were also shown in Figure 4E. *Proteobacteria* and *Methylobacterium* were enriched in the NACT group, whereas *Bacteroidetes*, *Firmicutes*, *Prevotella*, *Ruminococcus* and *Faecalibacterium* were enriched in the SURG group, all of which were key bacteria that segregated the intestinal microbiota in the NACT and SURG group.

## DISCUSSION

We conducted this study to explore the hypothesis that NACT could increase the incidence of postoperative infection in gastric cancer patients *via* damaging the intestinal barrier.

In our study, significantly higher incidence of infectious complications was shown in patients of the NACT group; this result is consistent with a previous study [8]. While the underlying mechanism of this phenomenon is still unclear, some reports suggested that damage of the immune system caused by using chemotherapy might be a key point, [9] and multiple patient and animal experiments had confirmed the changes [11–13]. However, studies focused on other localized changes, such as intestinal tissue damage caused by chemo drugs, were scanty. Although few animal models exhibited the mucosal toxicity of chemotherapy, [14] studies using human intestinal tissue to confirm these findings are relatively few.

We collected jejunum tissues of these patients during surgery to evaluate status of intestinal barrier. The most important structures in the intestinal barrier are TJs, which, accompanied by mucosal fluid, microvilli and other special structures, constitute the integral mucosal barrier. [15] In the present study, we found that the changes of the mucosa barrier including desquamation of epithelial cell, microvilli rupture and fusion, atrophy and edema of mucosa, disruption of intercellular TJs as well as mitochondria and endoplasm damages were shown in patients with infectious complications in overall. And similar morphological alterations in TJs have also been detected in patients who received NACT. Keefe et al. [16] found that there was an increase in number of open TJs within the small intestine of patients who received specific chemotherapy. Meanwhile, Dorner et al. reported that TJs damage increased maximal intestinal permeability of patients who received poly drug-chemotherapy [17]. S-1 and oxaliplatin (SOX) regimen, which was used in this study, included combination of S-1 and oxaliplatin, and cytotoxicity was one of the most important mechanisms for the anti-cancer effect of the two drugs. Considering the link between the integrity of TJs and normal intestinal barrier function as well as gut health, these studies, accompanied by ours, demonstrated that TJs disruption might play an important role in the gut toxicity caused by chemotherapy. In the meantime, TJs also functioned a barrier of epithelial cells. [18–20] More permeable than the transcellular pathway, the para-cellular pathway limits solute flow mainly by intact structure of TJs, while limited inflow of relatively large particles, such as bacterial lipopolysaccharides and proteins, can only be allowed through the leak pathway, although the size at which particles will not pass the leaky pathway has not been accurately defined, substances with similar dimension of bacteria are surely excluded. [21] Therefore, when TJs structure and permeability were compromised, it is likely that some intestinal pathological bacteria and viruses might break through the mucosa barrier and infect human body. Consequently, chemotherapy, *via* inflicting cytotoxic effects on intestinal epithelial cells, could cause damage to intestinal barrier junctions, consequently, a disrupted gut barrier allows pathogenic bacterium to invade the human body and cause a higher incidence of infectious disease in the NACT group.

Moreover, disordered expression of TJ proteins can also damage the TJ structure, disrupted protein-protein interactions, accompanied by cell polarity loss and barrier destruction, have been shown in several states of infections. [22] In the present study, the expression of claudin-1, occludin and ZO-1 in the intestinal mucosal significantly decreased in patients with infectious complications and those in the NACT group. Members of the claudin family are the most important transmembrane proteins, which contribute to several aspects of TJs permeability. Serious damage on organ function can be caused by inordinate expression of individual family members. The specific mechanisms of how occludin affects barrier function is still unclear, however these transmembrane TJ protein is able to interact with actin and claudins. ZO1, members of peripheral membrane proteins, are important to assembly and maintenance of TJs, which partly owns to their numerous domains which can interact with other TJ proteins, such as actin, occludin and claudins. [21] S-1, an orally 5-fluorouracil (5-FU) prodrug, contains tegafur, which can be continuously metabolized to 5-FU. Previous studies found that up-regulated nuclear factor kappa B (NF-kB), induced by 5-FU in the intestine, was able to enhance the expression of 5-FU-affected genes, [23] meanwhile activation of NF-kB have been observed in several inflammatory diseases, such as inflammatory bowel diseases (IBDs), glomerulonephritis and local joint inflammation, [24] besides multiple inflammatory cytokines, especially tumor necrosis factor (TNF), can induce TJs disruption. Therefore, the usage of 5-FU could result in an increase in intestinal permeability and a decrease in tight junction protein expression [25] which can also increase the para-cellular leakage that lead to the influx of bacterium and endotoxin in to the blood and higher incidence of infectious complications after surgery.

The changes of the bacteria barrier following the NACT was identified by 16S rRNA sequencing approach. Lower diversity was found in mucosal associated microbiota of patients who received chemotherapy. Reduced richness of the intestinal bacteria is a common feature of intestinal inflammation which was described in obese, old-age and IBD patients. [26, 27] Tissue samples of the NACT group were marked with increased abundance of *Proteobacteria* and *Methylobacterium*, and decreased *Firmicutes* (*Ruminococcaceae*), *Actinobacteria* (*Bifidobacterium*) and *Faecalibacterium*. In normal conditions, numerous aerobic and anaerobic micro-organisms colonize the lumen of the gut where a balance between probiotics and pathogens was formed to function in keeping metabolic homeostasis, adjusting inflammatory level and preventing pathologic bacteria from invading the system circulation. *Faecalibacterium*, and *Ruminococcus*, which are thought to be probiotics, are capable of lowering inflammatory level by adjusting the NFκB pathway. [28] *Bifidobacterium*, which was decreased in the NACT group, also possessed the ability to attenuate TNF-α and lipopolysaccharide induced inflammatory responses to inhibit inflammation in intestinal mucosa. [29, 30] Therefore, the decreased *Bifidobacterium*, *Faecalibacterium* and *Ruminococcus* could also modulate the intestinal permeability by inhibiting NFκB and TNF-α. *Bifidobacterium infantis* Y1 was capable of increasing the expression of TJs proteins and transepithelial resistance, [31] which might reduce intestinal permeability. Therefore, a decrease in *Bifidobacterium* might damage the function of mucosa barrier. Moreover, *Bifidobacterium*, *Faecalibacterium* and *Ruminococcus* are butyrate-producing bacteria [32] which help to maintain the integrity of mucus layer, because butyrate can increase synthesis of mucin *via* MUC2, [33] hence, the impaired mucus layer caused by chemotherapy-induced reduction of butyrate-producing bacteria might also lead to intestinal tissue damage and bacteria translocation. [34] Therefore, the changes of microbiota after NACT can also annihilate the function of intestinal barrier and lumen pathological micro-organisms as well as their toxic products can invade the human systemic circulation to cause remote infectious diseases.

One limitation of our study is that we detected bacteria that might be changed when patients received NACT, but the exploration of unknown mechanisms by which chemotherapy influence microbiota are surely needed by conducting animal or tissue based experiments in the future.

In conclusion, our study showed a higher incidence of postoperative infectious complications in patients who underwent NACT, and we used patients’ intestinal mucosal samples to demonstrate that intestinal barrier dysfunction, manifesting as damaged mucosal ultrastructure, lower expression of TJ protein and mucosal related dysbiosis, might be critical in this process. Therefore, our results will be useful in developing novel therapeutic methods focusing on maintaining intestinal barrier function to prevent postoperative infection. Furthermore, the deletion of probiotics after chemotherapy suggest that using probiotic agents might be helpful in reducing patients’ incidence of postoperative infectious complications.

## MATERIALS AND METHODS

### Patients

This cohort study recruited locally advanced gastric cancer patients from the general surgery department of Affiliated Hospital of Qingdao University, Qingdao, China between January 2013 and September 2014. We collected patients’ jejunum tissues during surgery and observed the incidences of post-operative infectious complications.

#### Inclusion criteria

The inclusion criteria for this study were age over 18 years, newly diagnosed and untreated, histological examination of adenocarcinoma of the stomach, tumor clinical stage of T4aN+M0 or T4bN+M0 based on endoscopic ultrasound (EUS) and contrast-enhanced CT (computed tomography) scan, capable of radical resection, hemoglobin above 90g/L, leukocyte count in excess of 3×10^9^ /L (absolute granulocyte count higher than 1.5×10^9^ /L), platelet count higher than 100×10^9^ /L, creatinine clearance values above 50 mL/min, liver enzyme levels no higher than three times control values and no major concomitant disease which might impact patients’ treatment strategy.

#### Exclusion criteria

The exclusion criteria were distant metastasis (M1), positive peritoneal cytology, carcinomatosis, cancer progression after NACT, history of other tumors, dihydropyrimidine dehydrogenase deficiency, and allergy to a chemo-drug. Moreover, patients whose cancer proved to be pathologic stage of T4b/N+M0 during surgery and received combined multiple organ resection were also excluded. Besides, the reconstruction of digestive tract for all patients included should be Roux-en-Y method, thus patients who received BI or BII method were excluded as well. Furthermore, in view of the microbiota nature of the analysis, patients who used antibiotics within 2 months before an operation, or were regularly using non-steroidal anti-inflammatory drugs, statins or probiotics were also excluded from the study. Other exclusions included chronic bowel disease, other signs of infections, food allergies and dietary restrictions.

Patients who received NACT treatment had been assessed by the multidisciplinary team of oncologists, surgeons, anesthetists and radiologists, and according to their EUS, CT, endoscopic biopsy, blood tumor marker tests results, their tumors might invade adjacent structures (T4b) and have to receive extended surgery (procedure which might include the greater and lesser omenta and any other organs involved by extension of the primary growth e.g., pancreas, spleen, mesocolon, colon, or left lobe of liver) to reach radical resection. And in order to reduce tumor size and burden, control microscopic disease, increase the likelihood of achieving an R0 resection and decrease the probability of adopting extended surgery, these patients received NACT treatment before surgery. Others, who received surgery first, were evaluated by the same team that their tumors might invaded serosa (T4a), and they were considered to benefit from surgery before chemotherapy. The number of cases in the area during the study period determined the sample size. Patients, who underwent radical resection right after diagnosis, were assigned to the SURG group, and those, who received NACT before and then underwent radical surgery, were assigned to the NACT group. Details of the treatment are stated in the following part.

### Preoperative treatment

Chemotherapy consisted of three courses (2-week administration and 1-week withdrawal) of S-1 at 40-60 mg bid/body per day (body surface area (BSA) < 1.25m^2^, 40 mg bid, 1.25 m^2^ ≤ BSA ≤ 1.5 m^2^, 50 mg bid, BSA > 1.5m^2^, 60 mg bid) accompanied by oxaliplatin at 130 mg/m^2^ on the first day of every course. Endoscopy with EUS and CT scan of the abdomen was conducted and blood cell count was obtained on the first day of each cycle. Responses were classified as complete response, complete disappearance of all lesions; partial response, more than 50% increase in the area after chemotherapy; stable disease, 0 to less than 50% increase in the area; and progressive disease, any decrease in the area and the appearance of new lesions. After three courses of treatment, patients were re-evaluated for the presence of potentially resectable disease by EUS, CT, endoscopic biopsy, blood tumor marker tests and symptom relief and underwent radical resection.

### Surgery

Surgery was scheduled to take place within three weeks after diagnosis of patients who directly received surgery treatment and three to six weeks after completion of the third cycle of chemotherapy of patients who had perioperative treatment. Laparoscopy-assisted gastrectomy under general anesthesia was adopted and performed by the same experienced surgeon at the general surgery department of Affiliated Hospital of Qingdao University, Qingdao, China. Surgery was guided by Japanese gastric cancer treatment guidelines 2014 (ver. 4) [35]. Generally speaking, in radical total gastrectomy, the whole stomach was removed, with the proximal line of division through the distal esophagus, and the distal line of division through the proximal duodenum. The procedure for a radical subtotal distal gastrectomy was the same, but a small, viable gastric remnant was left intact. In both procedures, the resection lines had to be at least 5 cm from the edge of the macroscopic tumor, when this rule could not be observed, frozen section was used to examine the resection margin. Lymph nodes resections strictly adhered to Standard D2 resection.

### Antibiotics usage and discharge

The antibiotics usage was guided by “Guidelines for Clinical Use of Antibacterials” released by the official website of National Health and Family Planning Commission of the People's Republic of China (http://www.moh.gov.cn/uploadfile/200410/200410912640959.doc). According to the guiding principle, the empirical antibiotics was used before we identified the types and drug resistance of pathogens, then specific antibiotic against pathogens would be given when the drug sensitive test result were confirmed. Antibiotics usage was stopped 3 to 4 days after the patients’ body temperature returned to normal and their symptoms were relieved. Besides, the principle of discharge, which adopted from the Japanese gastric cancer treatment guidelines 2014 (ver. 4), is initiation of solid food intake, removal of intra-abdominal drains and urinary catheter and stoppage of intravenous fluid administration.

### Sampling

The anastomosis method of Roux-en-Y were adopted to reconstruct the digestive tract of all patients included, during this procedure, the jejunum tissues 15 cm from the Treitz ligament were harvested, and fresh tissues were collected and stored at -80°C until use. The tumor pathologic stage were determined according to the American Joint Committee on Cancer system and all cancer specimens were graded histologically according to the World Health Organization classification criteria. Postoperative information was available for each patient studied. All the patients signed the informed consent prior to treatment and the study was approved by the Ethics Committee of Affiliated Hospital of Qingdao University.

### Infectious complications

Clinical definitions of postoperative infectious complications are listed in Table 6, and complications were rated using Clavien-Dindo classification system. [36]

**Table 6:** Clinical definitions of postoperative infectious complications

COMPLICATIONS	Clinical definitions
Systemic infection	
Bacteremia	7 day blood-cultures positive.
Pneumonia	A typical pulmonary infiltrate can be seen on a chest X-ray and/or the swab culture is positive.
Urinary tract infection	There are obvious symptoms including frequent micturition, urgency to urinate, and urodynia, accompanied by bacteriuria (100,000 cfu/mL).
Localized infection	
Incisional SSI	This infection occurs in the area of the skin where the incision was made or the incision area in muscle and the tissues surrounding the muscles.
Organ or space SSI	This type of infection can be in any area of the body other than skin, muscle, and surrounding tissue that was involved in the surgery. This includes a body organ or a space between organs. For example, anastomotic leakage.

SSI: surgical site infection.

### Histological measurement of intestinal mucosal injury

For histological examination, tissue samples were fixed in 4% paraformaldehyde, routinely processed, sectioned at 5 μm and stained with hematoxylin and eosin for light microscopic examination. The mucosal tissues were examined in a blind fashion by two independent pathologists. The degree of histopathological changes was graded semi-quantitatively using the histological injury scale previously described by Chiu et al [37] as follows: 0, normal mucosal villi; 1, development of a subepithelial space, usually at the apex of the villi with capillary congestion; 2, extension of the subepithelial space with moderate lifting of the epithelial layer from the lamina propria; 3, massive epithelial lifting down the sides of the villi and ulceration at the villous tips; 4, denuded villi with dilated capillaries and increased cellularity of the lamina propria; and 5, degradation and disintegration of the lamina propria, hemorrhage, and ulceration.

### Detection and observation of intestinal mucosal ultrastructure

The intestinal mucosal ultrastructure was characterized using transmission electron microscopy (TEM). For TEM assessment, a specimen of about 1 cm in length was excised with a sharp scalpel and fixed in 2.5% glutaraldehyde for 4 h at 4°C, followed by fixation in osmic acid and embedding in epon. Ultrathin sections were observed with a JEM-1200EX transmission electron microscope (Nippon Denshi Co., Tokyo, Japan) at an accelerating voltage of 1000 kV to detect ultrastructural injuries.

### Immunohistochemistry

Formalin-fixed and paraffin-embedded sections with a thickness of 4μm were deparaffinized, rehydrated and washed in phosphate buffered saline. The endogenous peroxidase activity was blocked with 3% H_2_O_2_ for 20 min and pre-incubated in normal goat serum for 20 minutes at room temperature. After blocking, the sections were incubated with primary anti-claudin-1, anti-ZO-1 and anti-occludin antibodies at 4°C overnight followed by incubated with biotinylated secondary antibody and then visualized with 3, 3′-diaminobenzidine. The staining results were scored by two pathologists blinded to the clinical data using the German immunoreactive score (IRS). Briefly, staining intensity was graded as “0” (negative), “1” (weak), “2” (moderate), and “3” (strong); staining extent was graded as “0” ( < 5%), “1” (5%-25%), “2” (25%-50%), “3” (50%-75%) or “4” ( > 75%). Values of the staining intensity and the staining extent were multiplied as a final IRS.

### PCR and 16S rDNA sequencing

PCR and 16S rDNA sequencing was performed as described previously [38]. Briefly, DNA was extracted from all samples using cetyltrimethylammonium bromide (CTAB) method with minimal modification. The PCR products were purified with AMPure XP beads (Agencourt Bioscience) to remove the unspecific products prior to library construction. Sequencing of qualified libraries was performed by the BGI-Huada Genomices institute (Shenzhen, China) using MiSeq System, with the sequencing strategy PE250 (PE251+8+8+251) or PE300 (PE301+8+8+301) (MiSeq Reagent Kit).

### Bioinformatics analysis

The sequences were clustered into operational taxonomic units (OTU) with a 97% threshold by using USEARCH (v7.0.1090), [39] and the OTU unique representative sequences were obtained. Chimeras were filtered out by using UCHIME (v4.2.40). [40] Representative OTUs were aligned to the optimized sequences and the abundance of OTUs per samples was obtained for performing further analysis. Ribosomal Database Project (RDP) Classifier v.2.2 was used to taxonomically classify OTU representative sequences in the following databases: Greengene V201305; [41] RDP (Release9 201203). [42]

### Statistical analysis

Continuous data are presented as mean ± standard deviation. The association between clinicopathological variables were examined by χ2 tests, Student t test and Mann-Whiteny test. The categorical data were analyzed by a Fisher's exact test, and the comparison of infectious complications between the two groups were analyzed by Mann-Whiteny test. We used Metastats (http://metastats.cbcb.umd.edu/) and R (v3.0.3) to determine which taxonomic groups were significantly different between groups of samples. Benjamini-Hochberg false discovery rate (FDR) correction was used to adjust the obtained P-value (function ‘P.adjust’ in the stats package of R (v3.0.3)). P values were two sided, and those < 0.05 were considered statistically significant. Analyses were performed with the SPSS software 17.0.
